# Excellent Self-Rated Health among Swedish Boys and Girls and Its Relationship with Working Conditions in School: A Cross-Sectional Study

**DOI:** 10.3390/ijerph18031310

**Published:** 2021-02-01

**Authors:** Sara Brolin Låftman, Maria Granvik Saminathen, Bitte Modin, Petra Löfstedt

**Affiliations:** 1 Department of Public Health Sciences, Centre for Health Equity Studies (CHESS), Stockholm University, SE-10691 Stockholm, Sweden; mia.granvik@su.se (M.G.S.); bitte.modin@su.se (B.M.); 2 Department of Public Health and Community Medicine, Sahlgrenska Academy, University of Gothenburg, Box 100, 40530 Gothenburg, Sweden; petra.lofstedt@folkhalsomyndigheten.se

**Keywords:** school demands, teacher support, classmate support, self-rated health, positive health, gender

## Abstract

The aim of this study was to investigate the extent to which school demands, teacher support, and classmate support were associated with excellent self-rated health among students, and to examine if any such statistical predictions differed by gender. Data were drawn from the Swedish Health Behaviour in School-aged Children (HBSC) study of 2017/18, performed among adolescents in grades five, seven, and nine (n = 3701). Linear probability models showed that school demands were negatively associated with excellent self-rated health, whereas teacher and classmate support showed positive associations. The link with school demands was stronger for girls than boys, driven by the finding that in grades five and nine, school demands were associated with excellent self-rated health only among girls. In conclusion, the study suggests that working conditions in school in terms of manageable school demands and strong teacher and classmate support may benefit adolescents’ positive health. The finding that the link between school demands and excellent self-rated health was more evident among girls than among boys may be interpreted in light of girls’ on average stronger focus on schoolwork and academic success. The study contributes with to knowledge about how working conditions in school may impede or promote students’ positive health.

## 1. Introduction

Adolescence is a life phase characterised by rapid development and increased autonomy. This period of life is also associated with various types of challenges, many of which are in relation to academic demands and social relationships in the family and among peers [1]. While adolescent health is important in its own right, health conditions in this period of life may also lay a foundation for the future. For instance, self-rated health in adolescence has been shown to be associated with health outcomes in adulthood [2,3]. Thus, research into the social determinants of adolescent self-reported health is vital. Prior research has presented links between adolescents’ self-rated health and a range of different medical, psychological, social, and lifestyle factors [4,5], including, e.g., healthy lifestyle [6], social capital in the family, neighbourhood, and school [7], exposure to bullying [8], and family socioeconomic position [9].

Focusing on the school as one central social context for adolescents, the current study examines experiences of school demands, teacher support, and classmate support, and their links with excellent self-rated health. There are several theoretical frameworks that aim to explain the role of working conditions for health among adults, two of the most influential ones being the demand–control–support (DCS) model and the effort–reward imbalance (ERI) model. The DCS model postulates that high demands in combination with low control and a lack of social support lead to higher levels of stress-related ill health [10], and the ERI model proposes that high effort combined with low reward at the workplace is connected with an increased risk of stress-related ill health [11]. Under the assumption that working conditions are a social determinant of health among students as well, both of these theoretical models have been applied to the school context. Studies have shown that high demands, low control, and a lack of social support at school were associated with higher levels of health complaints among students [12], and that experiencing effort–reward imbalance at school was related to a greater likelihood of reporting somatic complaints [13] and poor self-rated health [13,14].

In general, previous research on working conditions in the school setting and student health has largely focused on aspects of adverse health outcomes, including subjective health complaints [12,13,15,16,17,18,19,20], perceived stress [21], and conduct problems [16]. Yet, given schools’ potential to not only prevent health problems but also to promote health, it is important to examine the links between working conditions in school and students’ positive health as well, i.e., individuals’ subjective health and well-being, rather than the mere absence of health problems [22].

Prior studies have reported several types of school-related conditions to be associated with measures of positive health, such as subjective well-being, good self-rated health, and life satisfaction; students’ experiences of participation and good relations with teachers and peers have been linked with better health [23,24,25], whereas schoolwork pressure has shown inverse associations with health [24,26]. Taken together, however, there is a need for more studies investigating the relationships between students’ working conditions in school and positive health. Furthermore, since some studies have shown that the associations between working conditions in school and adverse health outcomes differ for boys and girls [15,19,20,21], it seems important to also take gender into consideration when examining the links between various aspects of students’ working conditions and positive health. Excellent self-rated health has been identified as an important component of positive health among adolescents [27], and is measured through a question about general self-rated health where the response category excellent is contrasted against the others [27,28]. The aim of the current study was to analyse the extent to which school demands, teacher support, and classmate support were associated with excellent self-rated health among adolescents in Sweden, and to examine if these associations differed by gender.

## 2. Materials and Methods

### 2.1. Data

Data were drawn from the Swedish Health Behaviour in School-aged Children (HBSC) study of 2017/18. The HBSC study was carried out among adolescents aged 11, 13, and 15 years, which in Sweden corresponds to grades five, seven, and nine. A two-step cluster sampling was performed. First, a random sample of schools were drawn, and thereafter, one class was randomly selected in each school. The response rate was 47% at the school level and 89% at the student level [29]. The total number of responding students was 4264. More information on the data collection is provided elsewhere [29]. The study sample was restricted to students with valid information on all study variables (*n* = 3701; 86.8%).

#### Measures

Excellent self-rated health was based on the question “Would you say your health is…?”, with the response categories “Excellent”, “Good”, “Fair”, and “Poor”. Students who responded “Excellent” were contrasted against all other categories, in line with prior research using self-rated health to measure positive health [27].

School demands were based on three items: “How pressured do you feel by the schoolwork you have to do?”; “I find schoolwork difficult”; and “I have too much schoolwork”. Response categories for the first question were: “Not at all” = 1, “A little” = 2.33, “Quite a lot” = 3.67, and “A lot” = 5. Response categories for the other two items were “Almost never” = 1, “Seldom” = 2, “Sometimes” = 3, “Often” = 4, and “Very often” = 5. Internal consistency was reasonably high (Cronbach’s α = 0.75). The two latter statements have previously been included in measures of school demands [16,17,18].

Teacher support was based on three items: “I feel that my teachers accept me as I am”; “I feel that my teachers care about me as a person”; and “I feel a lot of trust in my teachers”. The response categories were “Strongly agree” = 5, “Agree” = 4, “Neither agree nor disagree” = 3, “Disagree” = 2, and “Strongly disagree” = 1. Internal consistency was high (Cronbach’s α = 0.87). The same set of items have been used previously to measure teacher support [30].

Classmate support was measured by three items: “The students in my class(es) enjoy being together”; “Most of the students in my class(es) are kind and helpful”; and “Other students accept me as I am”. The response categories were “Strongly agree” = 5, “Agree” = 4, “Neither agree nor disagree” = 3, “Disagree” = 2, and “Strongly disagree” = 1. Internal consistency was high (Cronbach’s α = 0.80). The same set of items have been used previously to measure classmate support [30].

Measures of school demands, teacher support, and classmate support were formed by calculating the mean score of these scales if participants had responded to at least two out of three items within a scale. All scales ranged between 1–5.

Gender, grade, migration background (defined as being born abroad and/or having two parents who were born abroad), and socioeconomic position measured by the Family Affluence Scale (based on six items on the family’s number of cars, number of computers, number of bathrooms, ownership of a dishwasher, number of times travelled abroad on holiday in last year, and whether or not the student has his or her own bedroom [31]) were also included in the analyses.

### 2.2. Statistical Method

Linear probability models (LPMs) were performed, since the estimates can more readily be compared across models and across groups than in binary logistic regression analysis [32]. The tables present regression coefficients with 95% confidence intervals. To take into account the clustering of students in classes, robust standard errors were estimated. The number of classes was 213. First, a series of crude analyses were performed, including one independent variable at a time and controlling for gender and grade. Then, an adjusted analysis was carried out, mutually controlling for all independent variables. Next, interaction terms for gender with school demands, teacher support, and classmate support were included one at a time. Finally, adjusted analyses were performed separately for boys and for girls. R^2^ values are presented for the adjusted analysis of the total sample and for the gender-separated analyses. Analyses stratified by grade are presented in Appendix A.

## 3. Results

Descriptive statistics are presented in Table 1. In the study sample, 41.1% of the students reported excellent self-rated health. The sample was comprised of 49.1% boys and 50.9% girls, and of 26.6% students in grade five, 33.6% in grade seven, and 39.8% in grade nine. About one fourth had a migration background. The mean values were 2.87 for school demands, 4.10 for teacher support, and 3.93 for classmate support. The mean value of the Family Affluence Scale was 9.37.

Results from the linear probability models of excellent self-rated health are presented in Table 2. In the crude analyses, school demands were shown to be negatively associated with the probability of reporting excellent self-rated health, and teacher and classmate support showed positive associations.

The crude analyses also demonstrated that girls were 12 percentage points less likely than boys to report excellent self-rated health. Students in grades seven and nine were less likely to report excellent self-rated health compared with those in grade five (the differences corresponding to 9 and 15 percentage points, respectively). There was no difference in excellent self-rated health by migration background, whereas higher family affluence was associated with a greater likelihood of reporting excellent self-rated health.

In the adjusted analysis, all associations were attenuated, but (with the exception of grade seven in relation to the reference category) remained robust and statistically significant. The estimate of the migration background also turned positive and statistically significant at the 10% level. Subsequently, interactions between gender and school demands, teacher support, and classmate support were included one at a time. One statistically significant interaction was found, namely between gender and school demands (*p* = 0.005), indicating that the associations with excellent self-rated health were stronger among girls than among boys. The interactions between gender and teacher and gender and classmate support were, however, not statistically significant (teacher support: *p* = 0.120; classmate support: *p* = 0.747).

Finally, gender-stratified analyses were performed, with results presented in the right part of Table 2. These adjusted analyses show that school demands, teacher support, and classmate support, respectively, were associated with excellent self-rated health among boys and girls alike. However, as indicated by the interaction terms in the analyses of the total sample, the negative association with school demands was stronger for girls than for boys (for girls, the coefficient was −0.08, and for boys, the coefficient was −0.04).

Further analyses stratified by grade indicated that the gender difference in the relationship between school demands and excellent self-rated health was driven by students in (especially) grade five and in grade nine (see Table A1, Table A2 and Table A3 in Appendix A). In the gender-specific analyses of students in grades five and nine, respectively, statistically significant associations between school demands and excellent self-rated health were seen only among girls, whereas in grade seven, it was seen for both genders. Additionally, the statistically significant interactions between teacher support and gender and the gender-stratified models in Table A1 and Table A2 show that, among grade five students, teacher support was more strongly linked with excellent self-rated health among girls than among boys (Table A1), and that among grade seven students, an association between these two variables existed among girls, but not among boys (Table A2).

## 4. Discussion

Focusing on working conditions in schools and excellent self-rated health as a measure of positive health [27], this study showed a negative association between school demands and excellent self-rated health, and positive associations between teacher and classmate support and excellent self-rated health. These findings add to the body of research indicating that these kinds of school conditions are important for young people’s subjective health complaints [15,16,17,18,19,20,26], as well as for their inclination to report positive health [23,24,25,26] and school satisfaction [30]. In the current study, the associations between working conditions in schools and excellent self-rated health were observed for adolescents in general, albeit with some differences when breaking the data down by gender and grade.

First, school demands were more clearly associated with excellent self-rated health among girls than among boys, reflecting findings in prior studies showing that school demands were more strongly associated with adverse health among girls than among boys [15,19,20,21]. In the current study, school demands were clearly and inversely linked with health among girls in all grades, but for boys, this was true only among those in grade seven. One interpretation of this result is that girls, due to gender norms, tend to place greater emphasis on schoolwork and performance compared with boys [21,33], and that school demands therefore have greater implications for their positive health. Furthermore, this study showed gender differences in the relationship between teacher support and excellent self-rated health in grades five and seven. Among students in grade five, teacher support was more strongly linked with excellent self-rated health for girls than for boys, and among students in grade seven, an association between these variables was seen only for girls. These findings may potentially be interpreted in light of gender norms that encourage females to be interdependent and males to be independent [34]. While the effects of school demands and teacher support differed somewhat by gender and by grade in the current study, it is worth emphasising that the link between classmate support and excellent self-rated health was consistently strong and robust for both boys and girls across all grades, highlighting the fundamental importance of a positive social climate among peers at school for adolescent well-being [35].

Finally, the gender difference in excellent self-rated health also deserves to be stressed. Girls were significantly less likely than boys to report excellent self-rated health, which is consistent with prior cross-national research [28]. In the current study, this finding was driven particularly by students in grade nine, corroborating the results of a study based on earlier waves of Nordic HBSC data, which showed that the rates of students reporting excellent self-rated health were lowest among 15-year-old girls [27].

The main strength of this study is the large national sample that included information from nearly 4000 Swedish adolescents aged 11–15 years. However, there are limitations. First, it should be acknowledged that the relationships found between our independent and dependent variables are mere associations, and are adjusted for students’ sociodemographic characteristics only. As noted above, a broad range of medical, psychological, social, and lifestyle factors have been associated with adolescent self-rated health [4,5,6,7,8]. Some of these factors may possibly be related to working conditions in schools, and could thus be potential confounders in the reported associations. However, in the current study, we adjusted only for sociodemographic characteristics, since the inclusion of potential confounders should rest on previous theoretical or empirical grounds. Second, the cross-sectional design means that we cannot draw conclusions about causal relationships with support in the data. Hence, a relevant task for future research is to use longitudinal data with information on both school-related conditions and excellent self-rated health at several points in time. Such a study design would also be fruitful for examining possible mechanisms in these associations. Third, the relatively high non-response at the school level and the potential risk of systematic bias in the attrition means that generalisations to Swedish students in grades 5–9 should be made with caution. Furthermore, additional studies are needed to be able to generalise the findings to other educational and national contexts and to other age groups. Finally, it is also possible that there is systematic bias in the non-response at the student level due to absence from school on the day of the survey, although we are not able to judge if or how this could have affected our findings.

## 5. Conclusions

This study contributes with knowledge about how working conditions in schools may impede or promote students’ excellent self-rated health. The findings indicate that, in order to strengthen the prerequisites for positive student health, schools should support their students to make the demands placed on them manageable, ensure that teachers are given the time and resources to provide adequate support, and promote a positive social climate among students. Such features may be enhanced through targeted health promotion programmes, but also through broader efforts towards school effectiveness [36].

## Figures and Tables

**Table 1:** Descriptive statistics (*n* = 3701).

Variables	*n*	%	
Excellent self-rated health	1521	41.1	
Gender			
Boys	1818	49.1	
Girls	1883	50.9	
Grade			
Five	984	26.6	
Seven	1242	33.6	
Nine	1475	39.8	
Migration background			
No	2848	77.0	
Yes	853	23.0	
	Mean	s.d.	Range
School demands	2.87	0.93	1–5
Teacher support	4.10	0.86	1–5
Classmate support	3.93	0.77	1–5
Family Affluence Scale	9.37	1.97	0–13

**Table 2:** Results from the linear probability models of excellent self-rated health (*n* = 3701, distributed over 213 classes).

Variables	Crude ^a^		Adjusted ^b^		Boys ^b^		Girls ^b^	
	Coef.	95% CI	Coef.	95% CI	Coef.	95% CI	Coef.	95% CI
School demands	−0.10 ***	−0.12, −0.08	−0.06 ***	−0.08, −0.04	−0.04 **	−0.07, −0.01	−0.08 ***	−0.11, −0.05
Teacher support	0.11 ***	0.09, 0.13	0.06 ***	0.04, 0.08	0.05 **	0.02, 0.08	0.07 ***	0.04, 0.09
Classmate support	0.13 ***	0.11, 0.15	0.09 ***	0.07, 0.11	0.09 ***	0.06, 0.13	0.08 ***	0.05, 0.11
Gender								
Boys (ref.)	0.00	-	0.00	-				
Girls	−0.12 ***	−0.15, −0.08	−0.08 ***	−0.11, −0.05				
Grade								
Five (ref.)	0.00	-	0.00	-	0.00	-	0.00	-
Seven	−0.09 ***	−0.14, −0.04	−0.04	−0.09, 0.01	−0.06 ^†^	−0.12, 0.002	−0.02	−0.08, 0.05
Nine	−0.15 ***	−0.20, −0.10	−0.06 *	−0.11, −0.01	−0.04	−0.10, 0.02	−0.07 ^†^	−0.13, 0.003
Migration background								
No (ref.)	0.00	-	0.00	-	0.00	-	0.00	-
Yes	0.00	−0.04, 0.03	0.03 ^†^	−0.01, 0.06	0.08 **	0.04, 0.13	−0.02	−0.07, 0.03
								
Family Affluence Scale	0.03 ***	0.02, 0.04	0.03 ***	0.02, 0.03	0.03 ***	0.02, 0.04	0.02 ***	0.01, 0.03
								
R^2^	-		0.11		0.07		0.13	
								
Tests for interactions:								
Gender × school demands			−0.05 **	−0.08, −0.01				
Gender × teacher support			0.03	−0.01, 0.06				
Gender × classmate support			0.01	−0.03, 0.05				
								
*n*	3701		3701		1818		1883	

*** *p* ≤ 0.001, ** *p* ≤ 0.01, * *p* ≤ 0.05, ^†^ *p* ≤ 0.10, ^a^ including one independent variable at a time and controlling for gender and grade, ^b^ including all independent variables simultaneously.

## Data Availability

The Swedish HBSC data of 2017/18 can be applied for at the Public Health Agency of Sweden. Data from previous waves in Sweden and in other participating countries is available at: https://www.uib.no/en/hbscdata.

## Appendix A

**Table A1:** Results from the linear probability models of excellent self-rated health among grade five students (*n* = 984, distributed over 65 classes).

Variables	All ^a^		Boys ^a^		Girls ^a^	
	Coef.	95% CI	Coef.	95% CI	Coef.	95% CI
School demands	−0.07 **	−0.12, −0.03	−0.03	−0.09, 0.03	−0.13 ***	−0.19, −0.06
Teacher support	0.09 **	0.03, 0.14	0.07 *	0.01, 0.13	0.12 **	0.04, 0.21
Classmate support	0.09 **	0.04, 0.15	0.09 **	0.03, 0.15	0.09 *	0.01, 0.18
Gender						
Boys (ref.)	0.00	-				
Girls	−0.05 ^†^	−0.12, 0.01				
Migration background						
No (ref.)	0.00	-	0.00	-	0.00	-
Yes	0.06 ^†^	−0.01, 0.14	0.16 **	0.07, 0.25	−0.06	−0.19, 0.06
Family Affluence Scale	0.02*	0.0005, 0.03	0.02	−0.004, 0.04	0.01	−0.01, 0.04
R^2^	0.08		0.07		0.13	
Tests for interactions:						
Gender × school demands	−0.10*	−0.18, −0.02				
Gender × teacher support	0.08*	-0.0001, 0.16				
Gender × classmate support	0.04	−0.04, 0.12				
*n*	984		515		469	

*** *p* ≤ 0.001, ** *p* ≤ 0.01, * *p* ≤ 0.05, ^†^ *p* ≤ 0.10, ^a^ including all independent variables simultaneously.

**Table A2:** Results from the linear probability models of excellent self-rated health among grade seven students (*n* = 1242, distributed over 70 classes).

Variables	All ^a^		Boys ^a^		Girls ^a^	
	Coef.	95% CI	Coef.	95% CI	Coef.	95% CI
School demands	−0.08 ***	−0.11, −0.05	−0.09 ***	−0.13, −0.04	−0.08 **	−0.13, −0.04
Teacher support	0.03 ^†^	−0.01, 0.07	0.00	−0.06, 0.05	0.07 **	0.02, 0.11
Classmate support	0.07 ***	0.03, 0.11	0.07 *	0.004, 0.13	0.07 **	0.02, 0.12
Gender						
Boys (ref.)	0.00	-				
Girls	−0.04	−0.09, 0.02				
Migration background						
No (ref.)	0.00	-	0.00	-	0.00	-
Yes	0.01	−0.05, 0.07	0.04	−0.05, 0.13	−0.02	−0.11, 0.06
Family Affluence Scale	0.02 **	0.01, 0.03	0.02*	0.001, 0.04	0.02*	0.003, 0.04
R^2^	0.07		0.04		0.09	
Tests for interactions:						
Gender × school demands	−0.02	−0.09, 0.05				
Gender × teacher support	0.07 *	0.004, 0.14				
Gender × classmate support	0.04	−0.04, 0.12				
*n*	1242		610		632	

*** *p* ≤ 0.001, ** *p* ≤ 0.01, * *p* ≤ 0.05, ^†^ *p* ≤ 0.10, ^a^ including all independent variables simultaneously.

**Table A3:** Results from the linear probability models of excellent self-rated health among grade nine students (*n* = 1475, distributed over 78 classes).

Variables	All ^a^		Boys ^a^		Girls ^a^	
	Coef.	95% CI	Coef.	95% CI	Coef.	95% CI
School demands	−0.03 *	−0.06, −0.001	−0.01	−0.05, 0.03	−0.05 *	−0.10, −0.01
Teacher support	0.06 ***	0.04, 0.09	0.08 **	0.03, 0.12	0.05 *	0.01, 0.09
Classmate support	0.09 ***	0.06, 0.12	0.11 ***	0.06, 0.16	0.08 ***	0.04, 0.12
Gender						
Boys (ref.)	0.00	-				
Girls	−0.14 ***	−0.19, −0.08				
Migration background						
No (ref.)	0.00	-	0.00	-	0.00	-
Yes	0.02	−0.03, 0.08	0.06 ^†^	−0.01, 0.13	−0.01	−0.08, 0.07
Family Affluence Scale	0.04 ***	0.03, 0.05	0.05 ***	0.03, 0.07	0.03 **	0.01, 0.04
R^2^	0.14		0.12		0.10	
Tests for interactions:						
Gender × school demands	−0.03	−0.08, 0.02				
Gender × teacher support	−0.03	−0.08, 0.03				
Gender × classmate support	−0.04	−0.09, 0.02				
*n*	1475		693		782	

*** *p* ≤ 0.001, ** *p* ≤ 0.01, * *p* ≤ 0.05, ^†^ *p* ≤ 0.10, ^a^ including all independent variables simultaneously.
